# Assessment of left ventricle preload by transthoracic echocardiography: an easy task?

**DOI:** 10.1186/s40560-015-0090-7

**Published:** 2015-05-14

**Authors:** Pablo Blanco, Takako Sasai

**Affiliations:** Intensive Care Unit, Hospital Dr. Emilio Ferreyra, Necochea, 7630 Argentina; Department of Anesthesiology, Okayama Red Cross Hospital, 2-1-1 Aoe, Kita-ku, Okayama, 700-8607 Okayama Japan

**Keywords:** Central venous pressure, Left atrial pressure, Left ventricle preload, Transthoracic echocardiography, Extravascular lung water

## Abstract

In sicker hearts, right atrial pressure (an estimation of right ventricle preload) are not equivalent to left atrial pressure (an estimation of left ventricle preload). Both right and left atrial pressures are frequently estimated using invasive techniques and also transthoracic echocardiography. While right atrial pressure is easy to obtain with transthoracic echocardiography, the assessment of left ventricle preload or filling pressures is not simple. In relation to the study of Sasai et al. (J Intensive Care 2(1):58, 2014), this paper discusses in a succinct manner how to think and assess the left ventricle preload by transthoracic echocardiography.

## Correspondence

Pablo Blanco

Dear Editor,

In failing hearts (septic and non-septic etiology), right atrial pressure (RAP) which infers right ventricle (RV) preload is commonly not equivalent to left atrial pressure (LAP), which infers the left ventricle (LV) preload or filling pressures.

As a daily example, it is not surprising to see patients presenting with cardiogenic pulmonary edema, which denotes a high LAP value, with normal inferior vena cava (IVC) analysis, which indicates a normal RAP.

RAP is commonly obtained by central venous pressure (CVP) measurement or through the analysis of the IVC by transthoracic echocardiography (TTE) in spontaneous breathing patients. LAP is estimated with the wedge pressure (pulmonary artery catheter) or commonly with a set of TTE two-dimensional and Doppler parameters.

At respect and as expected, the study carried out by Sasai et al. [1] shows no correlation between CVP and some selected TTE parameters of left chamber size and function for estimating the LV preload. Although the authors’ efforts to promote their findings are commendable, some points should be addressed.

The set of TTE parameters used for comparison with CVP, although simple and repeatable, are probably not accurate parameters of LV preload. For example, a patient with dilated cardiomyopathy and depressed LV systolic function could also present with hypovolemia. The simple inspection of the LV and LA suggests an elevated preload; however, other parameters like transmitral inflow and mitral annular velocities may aid in the correct distinction. Also, a patient with RV systolic failure and a low RV stroke volume, like in some cases of septic cardiomyopathy, also accompanied by several grades of tricuspid regurgitation, can have an elevated RAP, with normal or low LAP (again, the mitral inflow and annular parameters also aid in the correct diagnosis).

These are some examples that suggest that more reliable parameters of LV filling are necessary along with two-dimensional measurements.

**Fig. 1:**
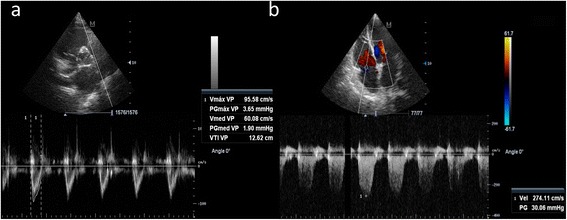
Example for estimating the pulmonary vascular resistance. **a**. Right ventricle outflow tract velocity time integral (12.6 cm) obtained in parasternal short-axis view. **b**. Right ventricle to right atrial peak velocity (2.7 m/s) obtained from a tricuspid regurgitation jet in apical four-chamber view. PVR is obtained as (2.7 /12.6) × 10 + 0.16, equal to 2.3 Wood units (normal <1.5)

With respect to tricuspid regurgitation, this jet allows to measure the pressure gradient between the RV and the RA (RV–RA pressure gradient). The increase of this gradient usually indicates the elevation of the right ventricle systolic pressure (or in practice the pulmonary artery systolic pressure, PAsP, in the absence of RV outflow stenosis). As recommended in guidelines [2], PAsP is an important data to obtain when LV filling pressures are investigated, nevertheless, pulmonary arterial hypertension (PH) may develop secondary to a pre-capillary, post-capillary, or the coexistence of both causes. High-flow PH can also occur for example in patients with high cardiac output (e.g., sepsis). Although not adequately established to be recommended for routine use, RV afterload (i.e., pulmonary vascular resistance (PVR)) can be estimated by TTE as RV–RA peak velocity (in m/s) divided by RV outflow tract (RVOT) velocity–time integral (VTI, in cm). PVR is estimated using the formula (RV–RA gradient/RVOT VTI) × 10 + 0.16 (Wood units). Normal PVR is <1.5 Wood units [3] (Fig. 1). This determination may aid in discrimination between high-flow PH (normal PVR) and non-high flow PH (high PVR). Additionally, a high PVR can be inferred when a short pulmonary artery acceleration time and a mid-systolic notch is evident in pulmonary artery Doppler spectrum.

**Fig. 2:**
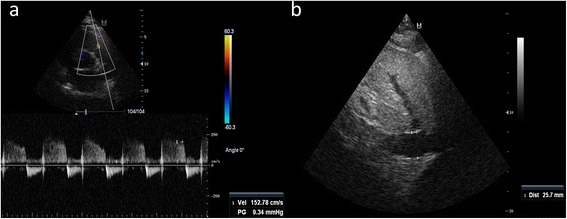
Example for estimating the pulmonary artery diastolic pressure (PAdP). **a**. The end-diastolic velocity of the pulmonary regurgitation jet was measured (parasternal short-axis view) and pressure gradient was automatically calculated (9.34 mmHg). **b**. Right atrial pressure was estimated in 15 mmHg through the analysis of size (dilated, 25.7 mm) and collapsibility (diminished) of the inferior vena cava. PAdP is obtained as the sum of 9.34 and 15 mmHg, resulting in a highly elevated PAdP (equivalent to an elevated left atrial pressure), of 24.3 mmHg

Pulmonary artery diastolic pressure (PAdP) which is derived from the sum of RAP and the end-diastolic velocity of the pulmonary regurgitation (Fig. 2) has shown a good correlation with LAP and thus is a valuable alternative for assessment of LV filling pressures [2]. In the absence of extreme elevations in PVR, a PAdP higher than 12 mmHg is indicative of a high LAP.

**Fig. 3:**
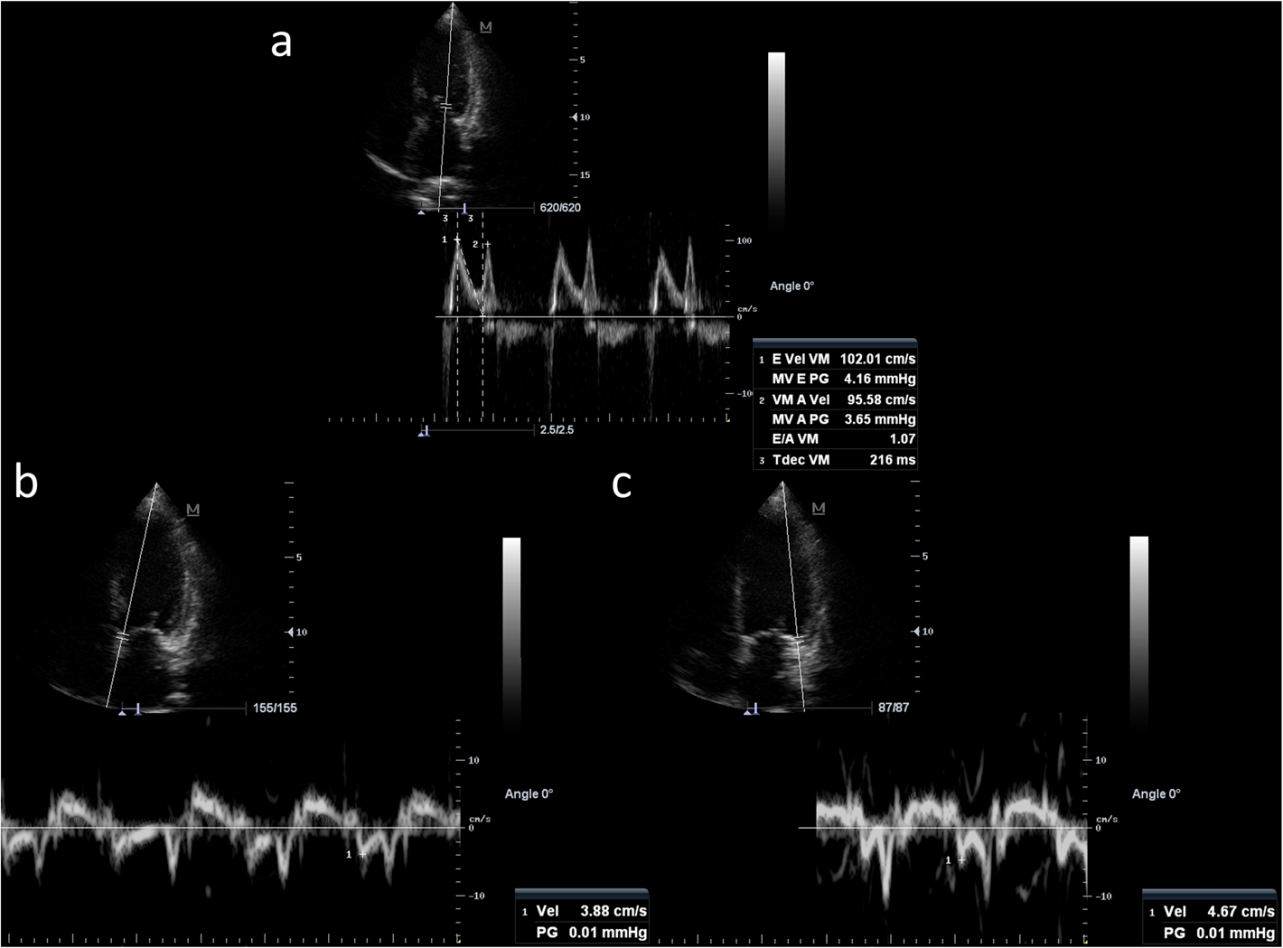
Example for estimating the left ventricle filling pressures from transmitral flow **(a)** and mitral annular velocities (septal in **b** and lateral in **c**), obtained in apical four-chamber view. The E velocity is 102 cm/s and the averaged annular velocity (septal and lateral/2) is 4.27 cm/s, with E/Ea ratio of 23.8, indicating a severe elevation of filling pressures

In concrete, estimating LV preload with echocardiography is not easy and at least some measurements are necessary along with two-dimensional images, such as transmitral flow pattern (E velocity, A velocity, E/A ratio, E deceleration time), mitral annular velocities (averaged septal and lateral velocities, Ea), and most importantly, the relationship between E and Ea (E/Ea) (Fig. 3). PAsP and PAdP can also be used as a surrogate of LAP and LV filling pressures. Along with the aforementioned parameters, the assessment of extravascular lung water with lung ultrasound is another useful parameter to add when evaluating LV preload.

### Response

Takako Sasai

Dear Editor,

We thank Dr. Blanco for his comments on our article.

In our study, we revealed that transthoracic echocardiography (TTE) findings are more informative marker for fluid management than CVP findings for assessing the left ventricular (LV) preload of patients with septic shock. LV preload is defined as a tension on the LV myocardium at the end of the diastolic phase (end-diastolic wall stress) and is clinically defined as LV end-diastolic volume (LVEDV) or LV end-diastolic pressure (LVEDP). In our study, we used LV end-diastolic diameter (LVEDD) as an index for LV preload. However, the authors have mentioned that LV preload cannot be easily evaluated with a single parameter, and a more detailed investigation is required. In daily practice, TTE findings, including LVEDD, may be used for estimating LV preload when actual LVEDV or LVEDP values cannot be easily obtained. Practically, in individual cases, we should treat patients with many indices instead of a single indicator. A complete evaluation of LV preload is difficult to perform and is complicated. However, a simple measurement may be sufficient to make the right decision in emergency or intensive care. Our aim was to assess whether CVP is a reliable marker of left ventricular preload; therefore, we used LVEDD, a relatively easier to measure standard indicator of LV preload, as a substitute for LVEDV.

## Abbreviations

RAP: Right atrial pressure
RV: Right ventricle
LAP: Left atrial pressure
LV: Left ventricle
IVC: Inferior vena cava
CVP: Central venous pressure
TTE: Transthoracic echocardiography
PAsP: Pulmonary artery systolic pressure
PVR: Pulmonary vascular resistance
RVOT: Right ventricle outflow tract
VTI: Velocity-time integral
PAdP: Pulmonary artery diastolic pressure
